# Horizontal gene transfer is widespread in diverse eukaryotes

**DOI:** 10.1186/s12864-026-12958-7

**Published:** 2026-06-08

**Authors:** Kun Li, Fazhe Yan, Ziyao Feng, Pingping Zhang, Zhongqu Duan, Qingqiu Gong, David L. Adelson, Chaochun Wei

**Affiliations:** 1 School of Life Sciences and Biotechnology, Shanghai Jiao Tong University, 800 Dongchuan Road, Shanghai, 200240 China; 2 School of Biological Sciences, The University of Adelaide, Adelaide, SA 5005 Australia

**Keywords:** Horizontal gene transfer, Eukaryotes, Comparative genomics, Sequence composition bias

## Abstract

Horizontal gene transfer (HGT) is the transfer of genetic material between distantly related organisms. Although HGT is a pervasive mechanism of genetic exchange among prokaryotes, gene transfer events involving eukaryotes are generally considered rare and restricted to a small number of lineages. Here we report genome-wide identification of HGT regions (HGTs) in 10 eukaryotes, including human, mouse, cow, lizard, frog, zebrafish, fruit fly, nematode, *Arabidopsis* and yeast. By comparing their genomes with thousands of eukaryote, bacteria and virus genomes, we found between 10 and 237 non-redundant HGTs per eukaryote species. Third-generation sequencing across most of the 10 analyzed genomes, combined with targeted PCR in *Arabidopsis thaliana*, was applied to validate the result HGTs and exclude contamination. Genes impacted by HGTs are enriched in transmembrane transport. Some HGTs have duplicated extensively within the host genome, affecting hundreds, even thousands of genes. Our findings reveal that HGT is ubiquitous in all diverse eukaryotes analyzed here, and it is a non-negligible, previously underappreciated contributor to genome evolution for eukaryotes.

**Supplementary Information:**

The online version contains supplementary material available at 10.1186/s12864-026-12958-7.

## Introduction

Horizontal gene transfer (HGT) is the transfer of genetic material between organisms that is not from parent to offspring, and it is a major driver of genome evolution in bacteria and archaea [1]. On average, 81% of genes in bacteria were involved in HGT [2]. Recent evidence has shown that HGT events also exist in eukaryotes [3]. For example, HGTs have been reported from soil bacteria to the common ancestor of *Zygnematophyceae* and *embryophytes*, which increased its resistance to biotic and abiotic stresses during terrestrial adaptation [4]. HGT of a plant detoxification gene *BtPMaT1*, made whiteflies gain the ability to malonylate thus detoxing a common group of plant defense compounds [5]. Besides, 1,410 genes were acquired via 741 distinct transfers from non-metazoan donors in 218 insects and males lacking *LOC105383139* which was transferred from bacteria courted females significantly less in diamondback moths [6]. Another remarkable example of HGT is a ~ 1.5 Mb fragment of *Wolbachia spp.* DNA integrated into the pill bug *Armadillidium vulgare* genome, resulting in the creation of a new W sex chromosome [7] and fragments of the *Wolbachia* genome could also be transferred to other insects and nematodes [8]. HGTs have been observed in genomes of five parasitic plants in the *Orobanchaceae* family [9], unicellular pathogens [10] and blood-sucking parasites [11]. HGT events originating from endosymbiotic organelles were well-established [12], while some proposed that unicellular eukaryotes and early developmental stages are particularly vulnerable to HGT [13]. Mechanisms for the transfer of DNA into eukaryotic genomes have been described for viral infection, transposons, conjugation between bacteria and eukaryotes, or from endosymbionts (not only plastids and mitochondria) [14]. Some behaviors, such as predation, and lifestyles, such as parasitism, have been reported to promote DNA transfer in eukaryotes [15]. Therefore, while the prevalence of HGT may be rare in eukaryotes, compared to bacteria and archaea, it does occur. However, the scale and impact of HGT events in eukaryotes are largely unknown.

We presented here a computationally efficient method for identifying HGTs in eukaryotes using both sequence composition bias and sequence alignment, which we evaluated by a simulated dataset and by comparing with other reported results. Unlike previous approaches focused mainly on protein-coding regions only, our analysis was performed at the whole-genome level and enabled the detection of candidate HGT regions in both coding and non-coding regions of a genome. We applied this method to a set of 10 representative model organisms with high-quality genome assemblies, which is critical to minimizing contamination-driven false positives in genome-wide HGT detection. Many bacterial and viral genomes were also considered to reveal the potential media organisms or vectors for HGT events in eukaryotes.

## Results

### An HGT identification method for eukaryotic genomes and its evaluation

We created a computationally efficient method for detecting HGT regions in eukaryotic organisms, which extends beyond traditional protein-coding regions to comprehensively analyze non-coding regions as well. Since genome-wide HGT detection demands computationally intensive high-precision alignment (average amino acid similarity of reported HGTs is 39% [6]), we propose an optimized approach combining sequence composition screening with sequence alignment methods to achieve both computational efficiency and reliable detection (Fig. S1; see Methods).

In brief, we first identified genomic regions most different from the rest of the genome based on their k-mer frequencies and then we aligned these selected genomic regions to other genomes. Before sequence alignment, we divided all genomes into three different groups based on their taxonomic information, including self-group (SG), closely related group (CRG) and distantly related group (DRG), and more grouping information can be seen in Methods and Table S1. The genomic regions were considered as candidate HGT regions if their sequence conservation levels were discordant to the phylogenetic tree of relevant species. Specifically, a genomic region was determined as an HGT sequence occurred in the common ancestor of organisms listed in SG if it was more similar (in terms of alignment identity percentage and length) to genomes of species in DRG than those in CRG.

We evaluated the method with a simulated genome H containing simulated HGTs (see Methods). Since our HGT identification pipeline has two main steps (sequence composition-based screening and sequence alignment), the evaluation was done for both steps. While top 1% fragments with the largest differences with the host genome in terms of k-mer composition were input to the pipeline, 20.6% correct results would be identified after sequence composition-based screening and 17.77% correct results identified after sequence alignment (Fig. S2A; Table S2). When the percentage of fragments input was up to 20%, 58.9% and 57.1% correct results were identified after two steps respectively and it can be seen that the precision of prediction was higher than 90% for all cases (Fig. S2A; Table S2). This indicated that our pipeline improved processing speed without compromising the reliability of identified HGTs, albeit with a possible trade-off in recall.

We also evaluated our pipeline using the 170 HGTs previously reported for whitefly [6] (see Methods). When only the top 20% of genome fragments were input to our pipeline, 122 (71.8%) of the 170 previously reported HGTs were preserved after the sequence composition-based screening step, indicating that the first step retained most known candidate HGTs, and 55 (32.4%) of the 170 previously reported HGTs were identified after sequence alignment step (Fig. S2B). Because the overlap between the two datasets was not strictly one-to-one, these 55 previously reported HGTs corresponded to 62 HGT regions identified by our pipeline. Overall, our pipeline identified 131 HGTs, of which 62 (47.3%) overlapped with previously reported HGTs, whereas 69 (52.7%) did not (Fig. S2B; Table S3). Among the 69 HGTs not reported previously, 36 did not overlap with protein-coding sequences, making them inaccessible to previous HGT identification methods restricted to protein-coding regions. Of the remaining 33 newly identified HGTs that did overlap protein-coding regions, 24 covered less than 25% of the corresponding protein-coding sequence, which may explain why they were not detected previously. The remaining 9 showed higher coverage of the corresponding protein-coding regions (31.7%-100%), indicating that limited CDS coverage alone does not fully explain the non-overlap. For these cases, the discrepancy is more likely attributable to methodological differences between our nucleotide-sequence-based whole-genome pipeline and previous protein-sequence-based approaches, including differences in sequence type, alignment strategy, and filtering criteria (Fig. S2B). Of the 115 HGTs reported previously but missed by our method, 49 were left out by sequence composition-based screening step and the remaining 66 HGTs were not identified by our pipeline at the sequence alignment step. Among them, 35 had higher nucleotide sequence identity percentages with genomes in CRG than that in DRG while the other 31 had nucleotide sequence identity percentages lower than 50% with genomes in DRG (Fig. S2B).

The comparison with previously reported HGTs showed that our results were reliable and our method could identify some novel HGT events because it broke the restriction of identifying HGT regions involving protein coding regions only.

### HGT is ubiquitous in diverse eukaryotes

**Fig. 1:**
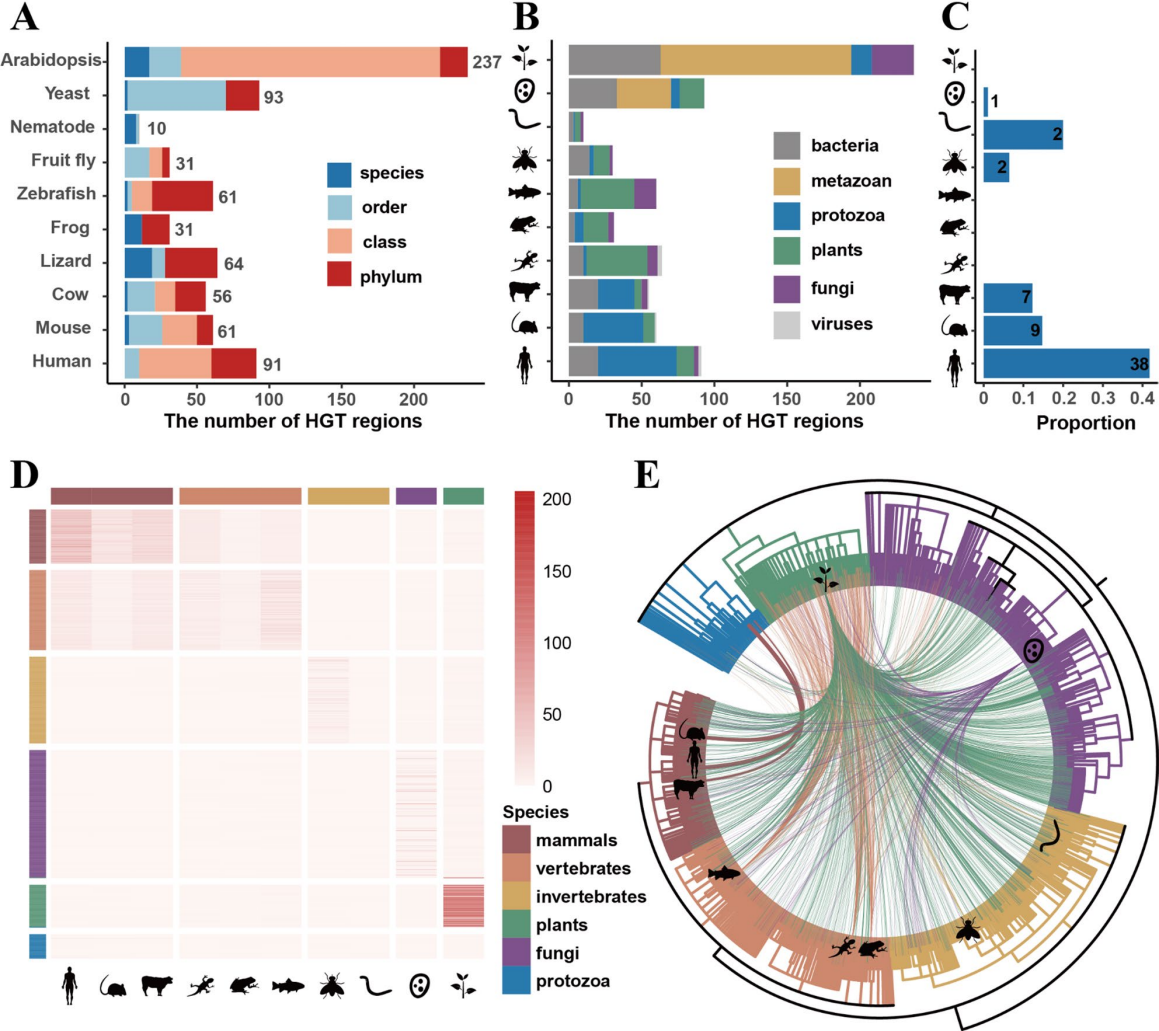
735 HGTs identified in 10 representative organisms. **A** The number of HGTs occurred in the common ancestors of organisms in a certain taxonomy level, such as species, order, class and phylum, which were defined as different SGs. **B** The number of HGTs with different HGT donor/acceptor organisms. **C** The proportion of HGTs reported previously [11, 16–22] among all predicted HGTs. The numbers on the bars are the absolute counts. **D** The HGT-appearance numbers between 10 representative organisms (X axis) and 1,496 eukaryotes (Y axis) are represented by the grid colors in the heat map. **E** HGTs between eukaryotes were shown by the lines connecting related species, and the thickness of the line represented the HGT-appearance number of the related species

We applied our HGT identification method to identify HGTs in 10 representative model organisms with high quality genomes from diverse eukaryotic lineages, including 1 primate, 2 mammals, 3 non-mammalian vertebrates, 2 invertebrates, 1 plant and 1 fungus. We identified between 10 and 237 non-redundant cross-kingdom eukaryotic HGTs for these 10 representative organisms, including human (*Homo sapiens*), mouse (*Mus musculus*), cow (*Bos taurus*), lizard (*Zootoca vivipara*), frog (*Xenopus tropicalis*), zebrafish (*Danio rerio*), fruit fly (*Drosophila melanogaster*), nematode (*Caenorhabditis elegans*), *Arabidopsis (Arabidopsis thaliana*) and yeast (*Saccharomyces cerevisiae S288C*). (Fig. 1A; Table S4). The number of HGTs found in nematode, an invertebrate, was the smallest, while the number for *Arabidopsis* was the largest (Fig. 1A). The HGTs of nematode are mostly species specific, while the HGTs of fruit fly and yeast mostly occurred in the common ancestor of organisms in the same order; human, mouse and *Arabidopsis* had more HGTs occurred in the common ancestor of organisms in the same class, while cow, lizard, frog, and zebrafish had more HGTs occurred in the common ancestor of organisms in the same phylum (Fig. 1A). The cross-kingdom species having the most similar sequence with the target organism was identified as the HGT related organisms. Overall, the HGT related organisms of these 10 representative species were distributed in 6 different kingdoms (bacteria: 183/731 [25.1%], metazoan: 168/731 [23.0%], protozoa: 154/731 [21.1%], plants: 153/731 [20.9%], fungi: 66/731 [9.0%], viruses: 8/731 [1.0%]) (Fig. 1B; Table S5). Bacteria, metazoan, plants, and protozoa occupied the highest proportion of HGT related organisms in mammals (human, mouse, and cow), non-mammalian metazoan (lizard, frog, zebrafish, fruit fly, and nematode) and non-metazoan eukaryotes (yeast and *Arabidopsis*) respectively (Fig. 1B). Especially, the *Actinomycetes* of bacteria, the *Insecta* of metazoan, the *Magnoliatae* of plants, the *Aconoidasida* of protozoa and the *Sordariomycetes* of fungi contained prevalent HGT related organisms (Fig. S3). Besides, most of the identified HGTs in the 10 representative organisms were previously unknown compared with reported HGTs [11, 16–22] (Fig. 1C).

To determine the frequency of HGT in eukaryotes, we calculated an HGT-appearance number N_AB_ for a representative organism A and another eukaryotic organism B, which was defined by the frequency with which organism B appeared in the phylogenetic trees of non-redundant HGTs of representative organism A. For instance, among the 91 non-redundant HGT trees for *Homo sapiens*, *Pan troglodytes* was found in 88 of them, therefore the HGT-appearance number N_AB_ between *Homo sapiens* and *Pan troglodytes* was 88. The distribution of HGT-appearance numbers N_AB_ between the 10 representative organisms and 1496 eukaryotes was shown in Fig. 1D and Table S6. The HGT-appearance numbers of organisms having the same taxonomic classification were higher (Fig. 1D), indicating that most HGTs identified by our pipeline were transferred before the divergence of model organisms and their sibling lineages. This also implies that these HGTs may have important functions since they have survived during the evolution [1]. By using this metric, we determined that 90.1% of eukaryotes (1348 of 1496) hosted HGTs (Fig. 1D and E), revealing widespread of HGT events across eukaryotes.

### Duplications of HGTs and their impact on their host genomes

**Fig. 2:**
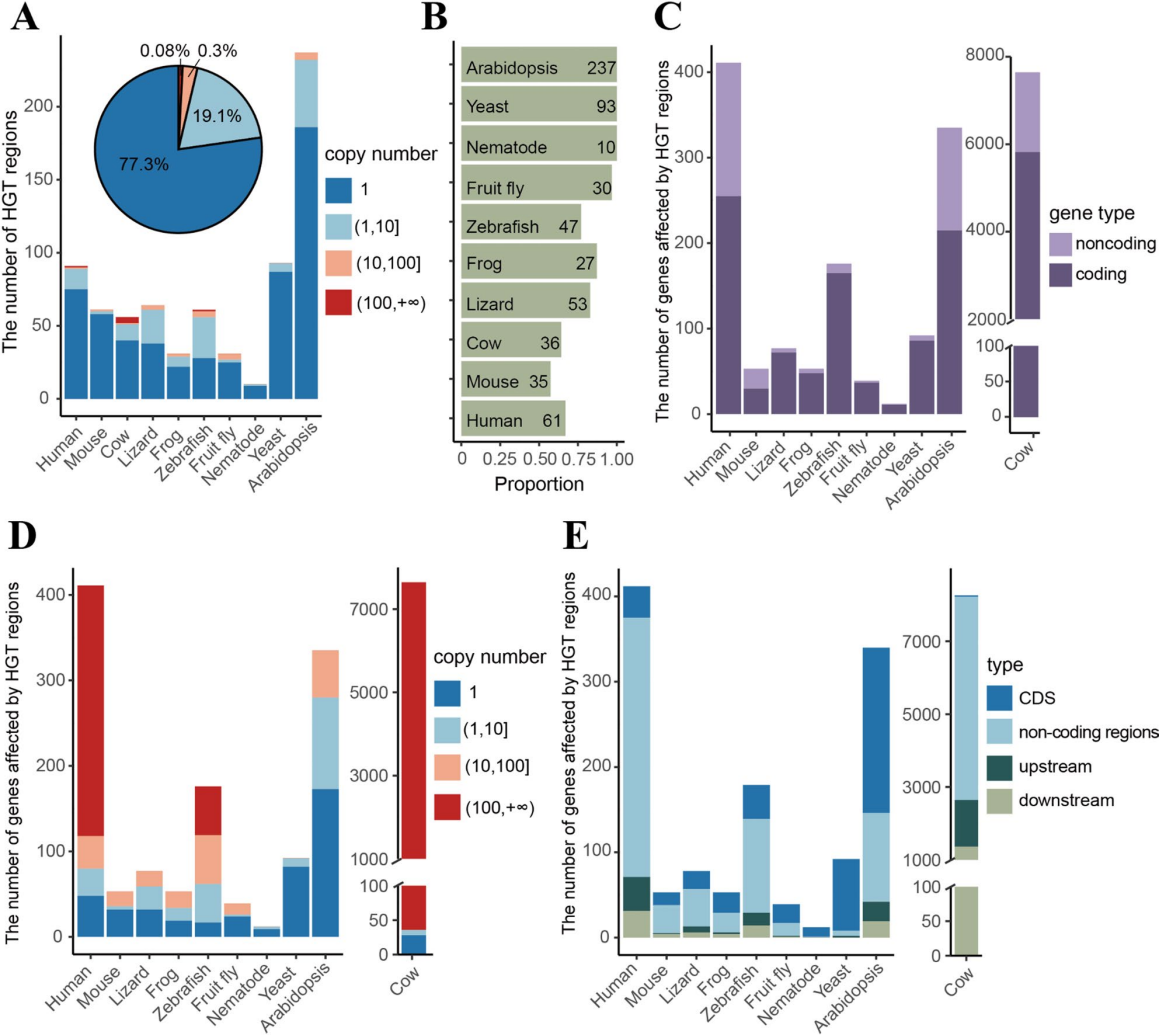
Duplications of HGTs and their impact on 10 representative organisms. **A** The composition of HGTs with different copy numbers. Although the number of unique HGTs were bigger, some HGT regions can have a huge number of duplications. **B** The proportion of HGTs overlapped with genes. While a majority of HGTs overlap with protein-coding regions across all species, this proportion is notably the lowest in the mouse. **C** The number of genes affected by HGT regions, not only in protein coding genes, but also in noncoding genes. **D** The number of genes affected by HGT regions with different copy numbers. Human, cow and zebrafish genomes have hundreds or even thousands of genes impacted by HGT duplications. **E** The number of genic elements affected by HGT regions. Most copies of HGTs overlapped with introns in vertebrates, while in invertebrates, fungi and plants, most of the HGT regions overlapped with CDS regions. After considering the lengths of introns and CDS regions, HGTs are more probable to overlap with CDS regions in invertebrates, fungi and plants compared with that in vertebrates (using Wilcoxon rank-sum test, *p* < 0.01)

We compared the non-redundant eukaryotic HGTs with their host genomes to further elucidate the impact of HGTs on their host genomes. Overall, 77.3% of HGTs (568 of 735) had a single copy in their host genomes, with the rest (22.7%, 167 of 735) had multiple copies, and 6 HGTs had more than 100 copies (Fig. 2A; Table S4). In particular, HGT region “NC_037357.1:83623202–83624198” related to BovB in the cow genome had 36,868 copies (with a total length of 36.3 Mbps), which was consistent with a previous study that BovB were present as many copies [16]. In newly identified HGTs, zebrafish HGT region “NW_018395302.1:126431–127344” had 126 copies and occupied 18 Kbps.

These HGT copies had affected many genes as well. In total, 85.6% of HGTs (629 of 735) had influence on 8,891 gene regions and the length of overlapped regions between HGTs and genes was shown in Figure S4. 75.8% of them were overlapping with protein coding genes (Fig. 2B and C). Especially, almost all HGTs impacted genes in *Arabidopsis*, yeast, nematode and fruit fly. There were 16 HGTs, each of which impacted more than 10 genes in their host genome (Table S4). For example, the zebrafish HGT region mentioned above and its copies overlapped (at least 1 bp) with 52 protein-coding genes (Fig. 2D), which was a huge impact on the zebrafish genome functions. HGTs with similar (but different degree of) impact on genome can also be found for most of the 10 representative organisms. Gene ontology (GO) analysis of genes affected by HGTs in different organisms shows that some were associated with synaptic membrane (human), actin cytoskeleton (mouse), ion transmembrane transport (cow), flavin adenine dinucleotide binding (fruit fly), organic hydroxy compound metabolic process (yeast), amino acid transport (*Arabidopsis*), etc. (Fig. S5; Table S7). The genes impacted by HGTs in the remaining 4 of the 10 representative species have no enriched functions.

The genes mentioned above were affected by HGTs in different genetic elements. As a whole, only 5.4% (485/8891) of genes affected by HGTs were affected in their CDS regions, with the rest were affected in the non-coding gene regions (UTRs and introns) and intergenic regions (especially those within 5,000 bps upstream of the gene regions and 5,000 bps downstream the gene regions) (Fig. 2E). This was because HGTs with a large copy number mostly affected the intron regions of the genes (Fig. S6), which might conform a rule for the occurrence of HGT, 'first do no harm' [1]. In detail, 75% (348/464) of genes affected by single-copy HGTs were affected in their CDS regions, while this proportion dropped to 0.2% (137/8427) for multi-copy HGTs and 71% (5982/8427) of genes affected by multi-copy HGTs were affected in their intron regions. Besides, the distribution of gene elements affected by copies of HGTs was different in different organisms (Fig. 2E). Most copies of HGTs overlapped with CDS regions in invertebrates, fungi and plants compared with that in vertebrates (using Wilcoxon rank-sum test, *p* < 0.01).

### Repetitive sequence composition of HGTs

**Fig. 3:**
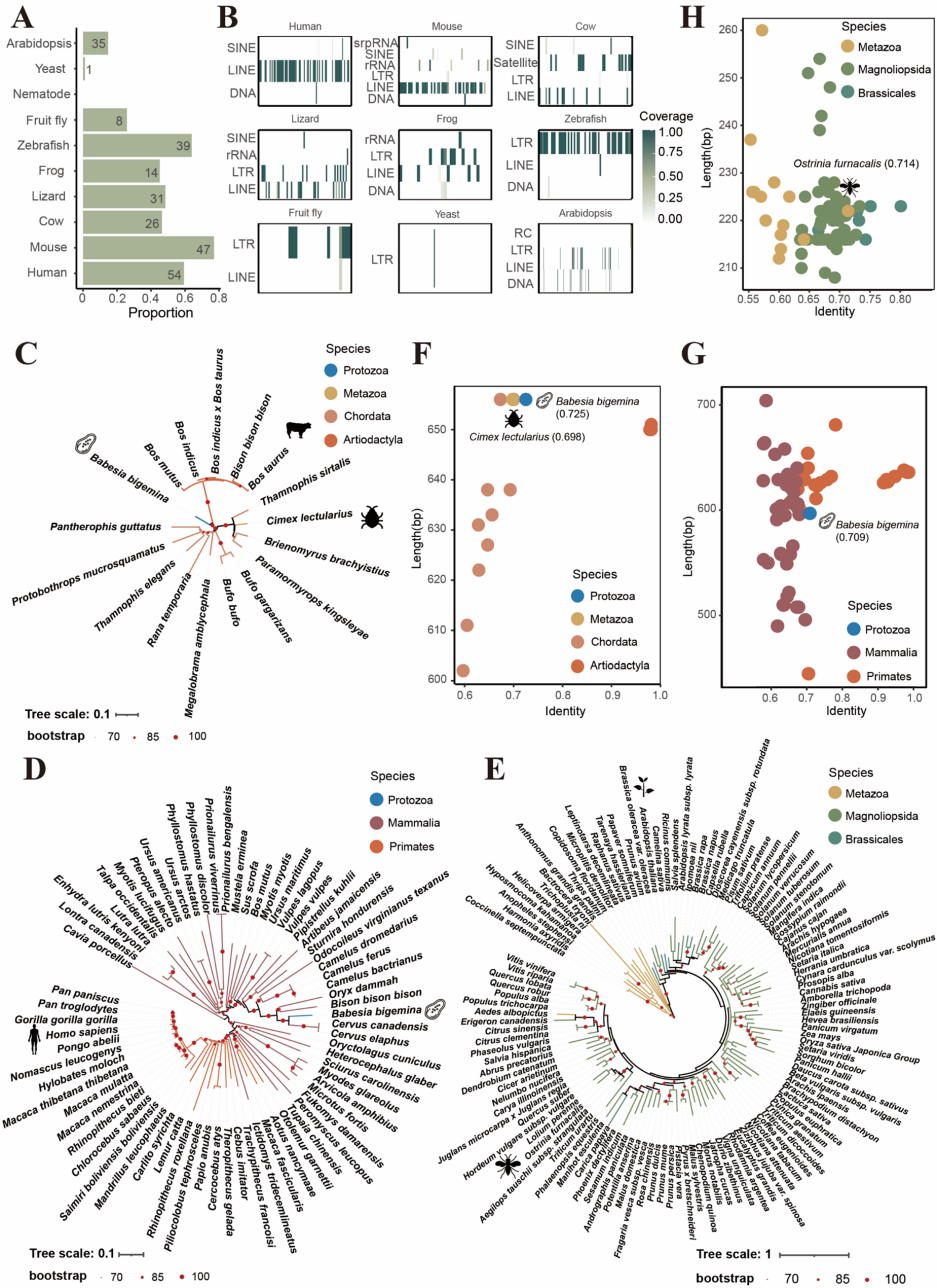
HGTs overlapping repetitive regions. **A** The proportion of HGTs overlapping with repeat sequences. Between 0 ~ 77% HGTs overlapped with interspersed repeats. **B** The proportions of different types of repeat sequences in each HGT across the whole genome. For each species panel, each column corresponds to a single, non-redundant HGT event, and each row corresponds to a specific type of repeats. The color intensity in each cell represents the length proportion of a specific repeat type within that particular HGT sequence. As shown, LINE retrotransposons were the most prevalent repeat type within HGTs detected in mammals, frogs, and lizards, while LTR retrotransposons were highly enriched within the HGTs of zebrafish, fruit flies, yeast, and *Arabidopsis*. **C**-**H** Phylogenetic trees and length-vs-identity plots of HGTs. The trees on left side represent the phylogenetic relationships of the homologous sequences of HGT regions. Only bootstrap values above 70 are shown. And the plots on right present sequence similarity between the homologous sequences from the model organism and the related species. **C**&**F** Cow HGT region “NC_037344.1: 42095551–42096199” overlapped with BovB retrotransposons; **D**&**G** Human HGT region “NC_000004.12:121865648–121866285” overlapped with L1 retrotransposons; and (E)&(H) *Arabidopsis* HGT region “NC_003076.8:12073716–12073960” overlapped with Gypsy LTR retrotransposons

We compared the non-redundant HGTs detected in 10 representative organisms with the repetitive sequences annotated in their reference genomes. Between 0% and 77% of their HGTs overlapped with interspersed repeats (excluding simple repeats) (Fig. 3A; Table S8), revealing significant species and repeat-specificity. The types of repeats overlapping with HGTs showed significant correlation with overall genomic repeat composition. Long interspersed nuclear elements (LINEs) retrotransposons were common in HGTs detected in mammals, frog and lizard, while long terminal repeat (LTR) retrotransposons were common in HGTs detected in fruit fly, yeast and *Arabidopsis*, consistent with their frequencies in their host genomes (Fig. 3B and Fig. S7). In comparison, the distribution of LTR in zebrafish HGTs was not consistent with their distribution in the host genomes, as seen the LTR in frog HGTs and the satellite in cow HGTs (Fig. 3B and Fig. S7). In the zebrafish genome, LTR appeared in as many as 37 non-redundant HGTs (60.7%), while that repeat only accounted for 4.2% of repeats in the genome.

BovB and L1 retrotransposons are highly prevalent in many eukaryotic genomes, especially in mammals [11]. The horizontal transfer of BovB is known to be widespread in animals [16] and horizontal transfer of L1 has been shown in plants, animals and several fungi [11]. In total, 4 of our non-redundant HGT events overlapped with BovB retrotransposons in cow (Table S9), supporting previous results for horizontal transfer of BovB [11, 16]. Furthermore, 119 L1 horizontal transfer events were identified in human, mouse, cow, lizard, frog and zebrafish (Table S9), providing more evidence that L1 elements are horizontally transferred [11].

All HGTs that overlapped with BovB retrotransposon were associated with the intracellular pathogen *Babesia bigemina* while one of them was also associated with the blood-sucking parasite *Cimex lectularius* (bed bug) (Table S9), both of which were the reported possible intermediary species [11]. *Babesia bigemina* is an Apicomplexan parasite that infects red blood cells [23], which infects livestock worldwide, including wild and domestic vertebrate animals. *Cimex lectularius* is known to feed on animal blood and can host over 40 zoonotic pathogens [24], thus transmitting many infectious diseases [25]. Figure 3C showed the tree of cow HGT region “NC_037344.1: 42095551–42096199” and its homologs. In addition to the two candidate vector species, this HGT tree also included 5 bovid mammals and 10 non-mammalian vertebrates (3 fishes, 3 amphibians, 4 reptiles) (Fig. 3C and F; Table S10), which were clearly clustered in distinct branches. Like in other parasites [26], it appears that *Cimex lectularius* and *Babesia bigemina* transfer DNA between the hosts it feeds on.

A considerable number of genes of intracellular pathogens have been acquired through HGT, including Apicomplexans [27]. Our analysis identified 76.5% (91/119) HGTs overlapped with L1 retrotransposons were associated with Apicomplexan intracellular pathogens, including *Babesia bigemina* and *Plasmodium vivax* (Table S9). For example, the tree of human HGT region “NC_000004.12:121865648–121866285” (Fig. 3D and G; Table S10) had shown the transfer between *Babesia bigemina* and mammals, including 26 primates, 11 artiodactyls, 11 carnivores, 9 rodents, 8 chiropterans, 1 eulipotyphlan, 1 lagomorph and 1 tree shrew.

Gypsy LTR retrotransposons are widely distributed among eukaryotes and have been found in plants, fungi and vertebrates [28]. The HGT of Gypsy is well known in *Drosophila* and plants, and has been found between fungi and non-seed plants [29]. In our results, 73 HGTs overlapped with Gypsy were identified in zebrafish, *Arabidopsis*, lizard, frog, fruit fly and yeast, which further supported previous reports. The *Arabidopsis* HGT region “NC_003076.8:12073716–12073960” showed the horizontal transmission of Gypsy LTR retrotransposons took place among 105 *Magnoliopsida* plants and 14 insects (Fig. 3E and H; Table S10). Some HGTs overlapping with repeat sequences of other 6 representative organisms were in Figure S8.

### HGTs overlapping with non-repeating sequences

**Fig. 4:**
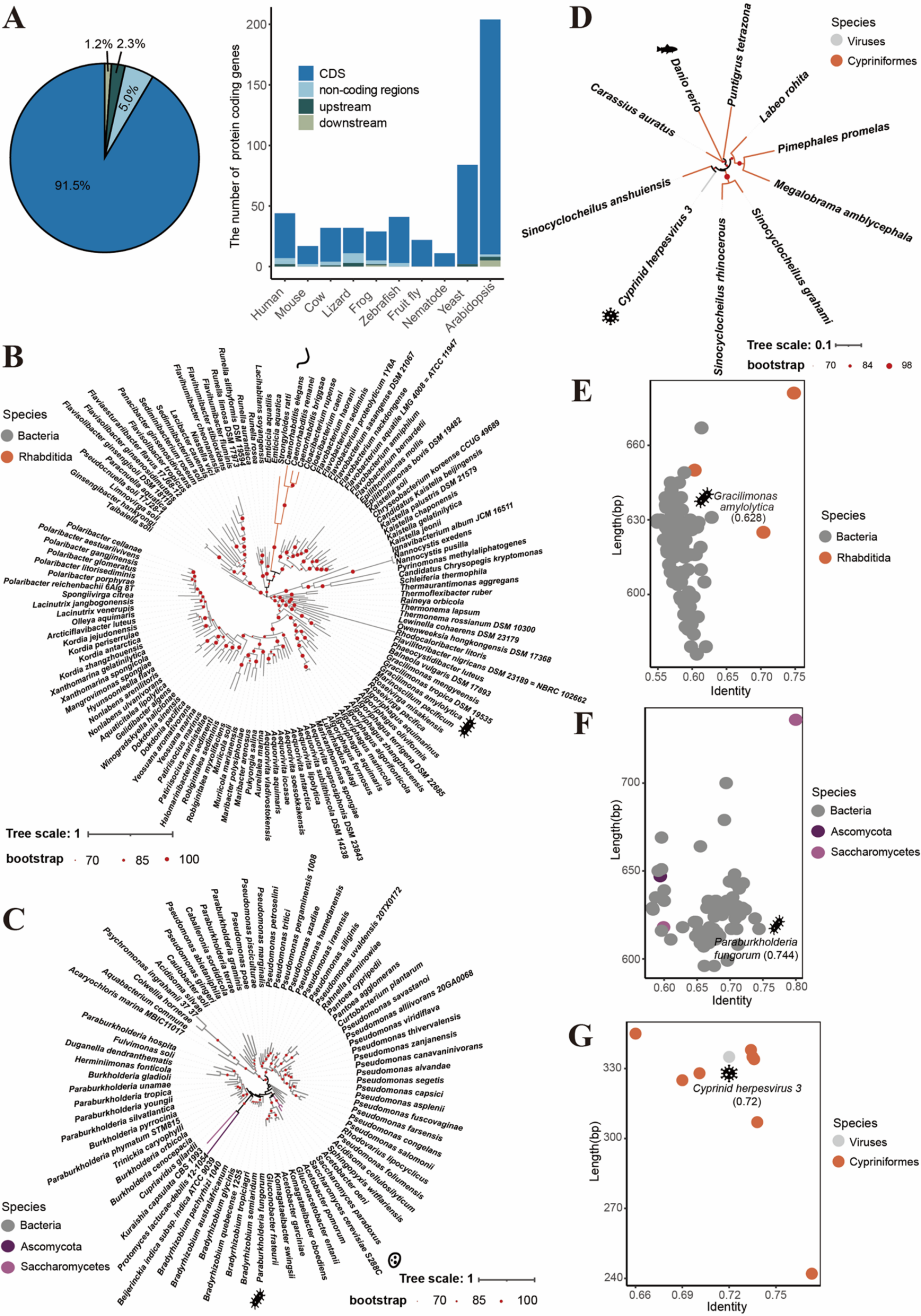
HGTs not overlapping repetitive regions. **A** The proportion of regions of protein coding genes affected by HGTs not overlapping with repeat sequences. Most protein coding genes affected by HGT events not overlapping with repeat sequences were affected on CDS regions. Phylogenetic trees and length-vs-identity plots of HGT regions not overlapping with repetitive regions. The trees on left side represent the phylogenetic relationships of the homologous sequences of HGT regions. Only bootstrap values above 70 are shown. And the plots present sequence similarity between the homologous sequences from the model organism and the related species. **B**&**E** Nematode HGT region “NC_003280.10:13635039-13635734” overlapped with gene CELE_C31C9.1 encoding a gut apical protein located in membrane raft; **C**&**F** Yeast HGT region “NC_001144.5:21121-21842” overlapped with gene YLL060C encoding Glutathione transferase GTT2; and **D**&**G** Zebrafish HGT region “NC_007123.7:45453300-45453643” overlapped with gene LOC562542, whose function is not clear

The above examples were mainly HGTs related to transposons and occurred in protozoa and eukaryotes. There were also 480 HGTs not overlapped with repetitive sequences (Fig. 3A; Table S8), and 424 HGTs (88.3%) of them overlapped with 516 protein coding genes (Table S11) while 91.5% (472/516) of protein coding genes affected by 424 HGTs were affected in CDS regions (Fig. 4A; Table S11). Gene ontology (GO) analysis of protein coding genes affected by HGTs in different organisms shows that some were associated with aldo−keto reductase activity (human), peroxisome (mouse), organic hydroxy compound metabolic process (yeast), amino acid transport (*Arabidopsis*), etc. (Fig. S9; Table S12). We did not find enriched functions for the protein coding genes impacted by HGTs in the remaining 6 of the 10 representative species. 

In order to better understand HGT events, we investigated several HGTs not overlapping with repetitive sequences. These HGTs were associated with bacteria and viruses. The HGT region “NC_003280.10:13635039–13635734” of nematode suggested the gene transfer between *Rhabditida* animals and bacteria (Fig. 4B and E; Table S10) and it overlapped with the CDS of gene *CELE_C31C9.1*, which encodes a gut apical protein located in membrane raft [30]. The yeast HGT region “NC_001144.5:21121–21842” overlapped with the CDS of gene *YLL060C*, having homologous sequences with *Ascomycota* fungi and *Pseudomonadota* bacteria (Fig. 4C and F; Table S10). Glutathione transferase GTT2 encoded by gene *YLL060C* is a novel atypical type of cytosolic Glutathione-S-transferases (GSTs), which are detoxification enzymes that catalyze the conjugation of electrophilic substrates to glutathione [31]. The HGT region “NC_007123.7:45453300–45453643” of zebrafish suggested the gene transfer between cyprinoid fishes and viruses (Fig. 4D and G; Table S10) and the HGT overlapped with the CDS of gene *LOC562542*, whose function is not clear. More trees of HGTs could be found in Table S13. Some HGTs not overlapping with repeat sequences but overlapping with CDS regions of protein coding genes of other 7 representative organisms were in Fig. S10-S17.

### Genes impacted by HGTs in *Arabidopsis thaliana* are enriched in transmembrane transport

**Fig. 5:**
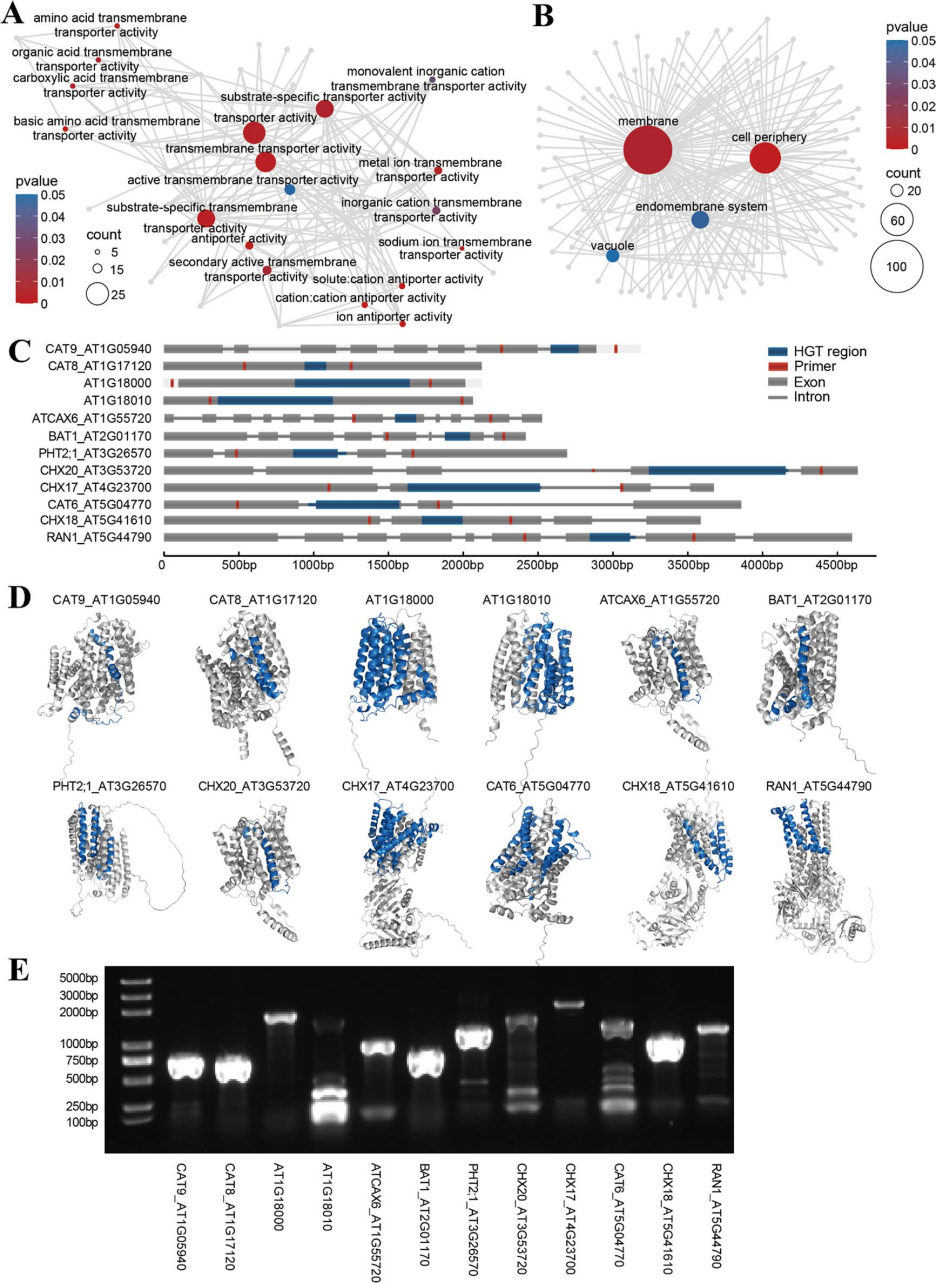
HGTs associated with transmembrane transport in *Arabidopsis thaliana*. **A**-**B** Gene ontology (GO) functional enrichment for genes affected by HGT events in *Arabidopsis thaliana* in terms of molecular functions (**A)** and cell components (**B).** Genes related to transmembrane transport were significantly enriched. The colored dots represent pathways and gray dots represent genes. The colors of colored dots represent the *p*-value and the sizes of colored dots represent the number of genes involved. **C** The structures of 12 genes overlapped with HGT regions which were transferred to *Arabidopsis thaliana* and associated with transmembrane transport. The blue rectangles represent HGT regions and the red rectangles represent primer pairs we designed for PCR validation of HGT regions. **D** Predicted protein structures of the 12 genes mentioned above. The blue parts correspond to HGT regions. **E** Agarose gel electrophoretic analysis of the PCR products of sequences including HGT regions transferred from bacteria or fungi to *Arabidopsis thaliana*. The corresponding full uncropped gel image is provided in Figure S18

We checked the protein-coding genes overlapped with HGT regions in *Arabidopsis thaliana* (see Methods for more details). Gene ontology (GO) analysis of these genes showed 23 protein-coding genes impacted by HGT regions were significantly enriched in transmembrane transporter activity and ion antiporter activity in terms of molecular function (Fig. 5A; Table S14). In terms of cell compartments, the most significant ones are cell periphery and membrane (Fig. 5B; Table S14). Among these 23 genes, 12 genes affected by 11 HGT regions were transferred from bacteria or fungi to the plants (Table S15). HGT regions were mainly located in the exon regions of these 12 genes (Fig. 5C). At the protein structure level, these HGTs showed structural preference for alpha-helical regions in general (6 out of 12 with *p* < 0.05, exact binomial test) (Fig. 5D, Table S16). This high density of helical structures, accompanied by minor connecting loops, indicates their important functions in membrane transport activities (Fig. 5D).

As mentioned above, gene ontology (GO) analysis of genes affected by HGTs shows that synaptic membrane was enriched in human and ion channel was enriched in cow (Fig. S5), both of which were also associated with transmembrane transporter activity. These suggested that horizontal gene transfer might play an important role in transmembrane transport in animals.

### Validation of HGT regions in *Arabidopsis thaliana* with targeted PCR and Sanger sequencing

We designed primer pairs for HGT regions involving protein coding genes in *Arabidopsis thaliana* and targeted PCR was executed to exclude the possibility of contamination (see Methods for more details). Among the 11 HGTs related to 12 genes encoding transporters which were considered as being transferred to plants, PCR experiment and Sanger sequencing revealed that all of them were indeed found in the genome of *Arabidopsis thaliana* (Fig. 5E, Figure S18, Table S17), thus excluding the possibility of contamination.

To physically validate the integration of HGTs and rule out false positives caused by reference genome misassembly or environmental contamination, we mapped third-generation sequencing (TGS) data to the 10 representative species (excluding lizard and frog). A candidate was considered successfully validated if the TGS coverage depth of the HGT region was continuous and not significantly lower than the depth of its immediate upstream and downstream flanking regions. An abrupt drop in HGT depth relative to the flanks strongly indicates an assembly artifact or scaffolded contaminant. Overall, 60.7–100% (average 90.3%) of the HGT candidates exhibited consistent depth profiles and were successfully validated (Table S18).

## Discussion

### Widespread footprint and terminology of eukaryotic HGT

In this study, we created a computationally efficient pipeline for identifying HGTs in eukaryotes by combining sequence composition bias and sequence alignment. A total of 735 HGT regions were identified across the 10 representative organisms. Furthermore, homologous sequences linked to these specific HGT events were detected in 90.1% of the eukaryotic species present within our reference database, suggesting that these genetic transfers have left a widespread evolutionary footprint across the analyzed lineages. Although the number of non-redundant eukaryotic HGTs per species, ranging from 10 to 237 regions per species, is relatively small compared to that in prokaryotes, our results unequivocally establish that HGT is ubiquitous in 10 diverse eukaryotic genomes analyzed here.

We reported horizontal transferred genomic regions including protein coding gene regions and non-coding regions. For those horizontal transferred regions located in non-coding regions, the term “horizontal gene transfer” may confuse a general reader. However, the term “Horizontal Gene Transfer (HGT)” is a historically developed consensus umbrella term for the non-genealogical transmission of nucleotide between organisms, not strictly limited to canonical protein-coding genes. In addition, the definition of a gene is also not limited to protein coding genes, such as non-coding genes widely used in literatures. Therefore, we use horizontal gene transfer throughout this paper. Furthermore, establishing the exact directionality of these transfers poses a critical challenge, as determining whether a eukaryotic host acts as the donor or the recipient requires deep, case-by-case topological resolution. To avoid unsupported assumptions regarding directionality, we have conservatively adopted the neutral phrasing “HGT involving” throughout this manuscript.

### Evolutionary and functional Impact

Most HGT regions identified by our pipeline were transferred before the divergence of the model organisms and their sibling lineages, which implied that these HGTs may have important functions as they have persisted. Overall, 22.7% of HGTs had multiple copies in their host genomes and these HGTs and their copies overlapped with 8,891 genes. Both of them indicate that HGT events affect not only genome size but also genome functions. However, only 5.4% of genes were affected by HGTs in their CDS regions with the rest affected in the noncoding regions (introns, UTRs of gene regions and intergenic regions). This result also indicates that it is reasonable to identify HGTs using genomic sequences, which can find some HGTs in non-coding regions compared to methods focusing on protein regions only.

### Mechanisms and potential transfer routes

Between 0 ~ 77% of HGTs overlapped with interspersed repeats, in which BovB, L1 and Gypsy retrotransposons had the highest frequency. By analyzing the phylogenic tree of those HGTs, we propose that blood-sucking parasites (*Cimex lectularius*) and intracellular pathogens (*Babesia bigemina*) are involved in BovB transfer between mammals and other vertebrates, while Apicomplexans (*Babesia bigemina* and *Plasmodium vivax*) are the hotspot of L1 transfer in eukaryotes, and Gypsy can be transferred between insects and plants. However, the routes for DNA transfer for the majority of HGT events in this report are still unclear.

### Methodological limitations

While our pipeline effectively identifies HGTs, several methodological limitations and trade-offs must be acknowledged. First, the parameter setting of our method involves a trade-off between computational efficiency and sensitivity. Under the default parameter setting (top 20% fragments), our method may omit ancient but ameliorated transfers, which can be avoided by setting the parameter to top 100%. With default parameter setting, it may lead to false negatives, omitting ancient, highly ameliorated transfers and occasionally missing shared HGTs among sister taxa. Second, because our criteria strictly targeted cross-kingdom transfers, intra-kingdom HGT events were excluded; thus, it is conceivable that the number of HGT regions is much larger than this. Third, although WGS depth profiling helps filter out misassemblies/contaminations by excluding regions located inside a contig with local low sequencing depth, it has limitations. To avoid discarding genuine HGT regions overlapping with repeat regions, we conservatively retained high-depth regions. This validation step can only filter out some of the contaminations which were assembled into the contigs during the genome sequencing of the target sequencing genome. If a contaminant were assembled into a separated contig in a genome assembly, this filtering step would not work. Finally, the existing reference genome collections showed uneven distribution of species coverage, potentially compromising the reliability of eukaryotic HGT distribution patterns derived from our study.

### Conclusion and future perspectives

With the fast progress of sequence technologies, many more eukaryotes, especially for those under-representative taxa, are going to have whole genome sequences, at high-quality or even T2T genome level quality. Eukaryote HGT identification and analysis study is opening a new door to the future. There is still much work to be done to better understand HGT. Although the overall number of events suggests HGT is a relatively minor source of novel genomic material compared to these fundamental mechanisms such as chromosomal rearrangements and transposable element activity, our findings reveal that HGT is ubiquitous in 10 diverse eukaryotic genomes. In conclusion, HGT is a non-negligible, previously underappreciated contributor of genome evolution for eukaryotes.

## Methods

In bacterial genomes, HGT regions are also called genomic islands (GIs) and can be detected using two distinct bioinformatics approaches, based on sequence composition or sequence alignment [32]. In general, the sequence composition of GIs is significantly different from that of the recipient genome. Composition-based methods identify GIs within genome sequences by calculating the k-mer frequencies of a fragment and comparing that frequency distribution with that obtained from the whole genome. Sequence alignment approaches are based on the premise that DNA sequence based phylogenetic tree topology of GIs will be discordant with respect to known species relationships, where sequences that are absent in several closely related organisms appear in more distant species. These two methods can be adapted to the identification of HGTs in eukaryotes but not without challenges. Due to the large sizes and the high heterogeneity of eukaryotic genomes, composition-based approaches may produce a number of false-positive predictions while sequence alignment methods are computationally expensive and time-consuming when hundreds of reference genomes must be aligned. In this study, we identified HGTs between eukaryotes and other organisms by combining these two approaches to reduce both the false-positive rate and computational cost.

### Data collection

The genome sequences were downloaded from NCBI RefSeq database (http://ftp.ncbi.nlm.nih.gov/genomes/refseq). All genomes were grouped into four datasets. The first dataset contained the reference genome sequences of 10 representative organisms consisting of 3 mammals, 3 non-mammalian vertebrates, 2 invertebrates, 1 fungus and 1 plant. The second dataset contained assembled genomes of 16,098 bacteria represent distinct species (no multiple genomes for a single species); the third dataset contained 11,695 viruses and the fourth dataset contained 1,496 eukaryotes including 193 mammals, 288 non-mammalian vertebrates, 312 invertebrates, 154 plants, 459 fungi and 90 protozoa. Detailed information about these genomes can be found in Table S19.

### Pipeline to identify HGTs

Figure S1 showed the pipeline to identify HGTs. Firstly, we identified genomic regions distinguishable from the rest of the genome based on k-mer frequencies. The selected genomic regions were then aligned with other genomes. Before sequence alignment, we divided all genomes into three different groups based on their taxonomic information, including self-group (SG), closely related group (CRG) and distantly related group (DRG). The self-group comprises all organisms descended from the specific common ancestor in which the HGT event originally took place. The genomic regions were considered as candidate HGT regions if their sequence conservation levels were discordant to the phylogenetic tree of relevant species. Specifically, genomic fragments were determined as HGT sequence occurring in the SG if they had higher identity percentage with species in DRG than that in CRG. Some similar sequences in DRG of candidate HGT regions were checked by alignment with whole genome sequencing (WGS) raw data of the same organism to avoid contamination artifacts. We then clustered the HGT sequences to obtain non-redundant HGTs. Phylogenetic trees for related species and homologous sequences were built for each HGT related species and homologous sequences of candidate HGT sequences.

### Sequence composition-based genomic fragments screening

Due to the large sizes of many reference genomes of model organisms, we first screened the potential genomic regions harboring HGT sequences. For each model organism, we split the genome sequences into 1000-bp segments with 200-bp overlapped regions across all chromosomes. Sequence segments with Ns were left out. Four-bp k-mer frequencies were obtained for the whole genome sequences as well as all genome segments. Euclidean distance was used to measure the difference between each segment and the whole genome sequence. All the distances were sorted in descending order. Finally, the fragments whose distances ranked in the top 10% for human, cow and mouse due to their larger genome sizes, top 20% for the other representative organisms were chosen for further analysis.

In the above statement, two parameters (k-mer size and fragments percentage) needed to be set. We chose appropriate parameters using 299 reported HGTs in the human genome [17], simple repeat sequences of which were filtered out using TRF (version 4.09) [33]. We tried different k-mer sizes (1 ~ 6), and k = 4 was selected because the highest portion of HGTs previously reported in the human genome were screened out in this situation (Fig. S19). A very high proportion (> 80%) of these human HGTs reported previously were kept in our results even if we only input top 5% of the fragments with highest differences to the human genome (Fig. S19).

### Sequence alignment

The sequence alignment was conducted using LASTZ (version 1.04.00) for the screened fragments of the model organisms and the whole genomes of other species with the following arguments: “--format = axt+ --ambiguous=iupac”.

### Re-screening the fragments and search for HGTs

Every target organism belongs to its own kingdom, phylum, class and order. For each classification level (phylum, class, order and species) of each representative organism, the other organisms were separated into three groups: self-group (SG) including all species in the same classification level with target organism, a closely related group (CRG) including all species in the same kingdom with target organism except those in SG, and a distantly related group (DRG) including all species in kingdoms without the target organism (Table S1). For example, when using class as the classification level and using human as the target organism, all mammals were regarded as SG, while the non-mammalian metazoan formed the CRG and DRG including all plants, fungi, protozoa, bacteria and viruses. We further screened the filtered fragments based on alignment results from LASTZ, to identify regions with discordant evolutionary relationships.

The aligned regions (ARs) of the input fragments were retrieved and used to identify putative HGTs. Firstly, we retained ARs aligned to DRG species if they met the following criteria: length > 135 bp, nucleotide identity ≥ 50%, and coverage ≥ 50%. For these ARs, we compared them with genomes in CRG, for which the identity percentage threshold was set to 50%. ARs were regarded as putative HGTs when they had higher sequence identity percentages with genomes in DRG than that in CRG.

To reduce false positive results generated by incorrect alignments, we removed ARs that contained the character ‘N’ or whose GC percentages were less than 0.3 or greater than 0.6. Finally, we checked the repetitive regions overlapping with ARs. RepeatMasker tracks were downloaded from the UCSC Genome Browser, or we ran *de novo* RepeatMasker [34] (version 4.0.7) (http://www.repeatmasker.org) to label the repeats of ARs. We then removed ARs that overlapped with simple repeats or low complexity repeats. We also applied TRF [33] (version 4.09) to remove ARs that overlapped with any tandem repeats. The remaining ARs were considered as candidate HGTs, and were used to build trees to determine discordance with known evolutionary relationships.

### Identifying non-redundant HGTs

HGTs were clustered using the cd-hit-est program (version 4.6.6) [35] with minimum nucleotide identity set at 80%. The longest sequences from each cluster were selected to represent the non-redundant HGTs.

### Validation of similar sequences in other kingdoms with WGS datasets

The power for detecting HGTs depended heavily on the quality of the reference genomes. Contaminating sequences from other species were the most likely sources of false positives. For the 10 representative organisms used in this study, their genome quality was considered very high, therefore the probability of sequencing contamination was negligible. However, the genome quality of other non-model organisms (such as parasites and protozoan pathogens) is often not high enough, which could cause false positive results. For example, if an abnormal HGT tree consists of only one parasite and several primates, and the process of constructing the reference genome of this parasite was contaminated by human DNA, this DNA transfer would be an artifact.

In order to avoid false positive HGTs caused by contamination, we checked the quality of genomes in DRG using whole genome sequencing (WGS) raw data of the same organism. Firstly, we calculated the number of genomes having potential homologous sequences of candidate HGT sequences in DRG. If the number was less than 10 and the kingdom eukaryote was used as the DRG, all the genomes containing potential homologous sequences would be checked with WGS data. In other words, if there were a sufficient number (> 10) of genomes supporting the existence of the potential homologous sequences, we assumed they were not derived from contamination. This value was set to 50 when the kingdom prokaryote was used as the DRG.

WGS raw data of the organism were downloaded from the SRA database. Due to the high computational demand of processing numerous WGS datasets, we employed a targeted alignment strategy. For each candidate sequence, we extracted its specific genomic region along with 1000 bp of upstream and downstream flanking sequences to construct a local reference index. The WGS raw reads were then aligned exclusively to these targeted regions to ensure accurate read anchoring. Subsequently, in order to rule out contamination or assembly errors, we calculated the sequencing depths and coverages of the potential homologous regions and their immediate 150 bp flanking regions. Sequence alignment was done using Bowtie2 [36] (version 2.2.4) and the calculation of sequencing depth was done using samtools (version 1.16.1) [37] with default parameters. The sequencing depth of homologous regions of HGTs should not be significantly lower than that of 150 bp upstream and downstream sequences of HGTs (using student’s t test, *p* > 0.05) and the sequencing depth of HGTs should be more than 10. Once a sequence met the above criteria within at least 2 samples, it was classified as not an artifact. The results were shown in Table S20.

### Exclusion of organellar DNA and Endosymbiotic Gene Transfers (EGTs)

To exclude Endosymbiotic Gene Transfers (EGTs) originating from the ancestors of mitochondria and plastids, the complete mitochondrial genomes of the 10 representative organisms and the *Arabidopsis thaliana* chloroplast genome were obtained from NCBI. We queried our non-redundant HGT candidates against these organellar reference genomes using BLASTN (e-value < 1e-5). Furthermore, we chose to exclude all sequences aligned to Cyanobacteria and Proteobacteria to ensure our final result is highly confident.

### Remove HGTs present in endogenous viruses

Endogenous retroviruses (ERVs) are widespread in vertebrates, making up nearly 8% of the genome of *Homo sapiens* [38]. Therefore, in order to avoid reporting sequences as HGTs that are actually from ancestral inheritance, we removed all HGTs found in ERVs. We collected ERVs from the repeat annotation of the UCSC genome browser, except for *Saccharomyces cerevisiae S288C* and *Arabidopsis thaliana*. All HGTs that overlapped with ERV genomic coordinates or aligned to ERVs using BLASTN (identity > 90% and length>100 bp) were removed.

### Construction of HGT phylogenetic tree

For each HGT, we searched for homologous sequences in other species based on the LASTZ output. When multiple regions in a species met the criteria, the best matched sequence, which had the maximal score weighted by the identity and multiplied by the alignment length, was picked to represent the homologous sequence. Based on the HGT sequence and the homologous sequences collected from other species, we ran multiple sequence alignment using MAFFT (version 7.520) [39] and then trimmed ambiguously aligned regions using trimAl (version 1.4.rev15) [40]. We then used the resulting alignment to infer the ML tree using IQ-TREE (version 2.2.2.3) [41] with its best-fitting model of amino acid evolution and 1000 ultrafast bootstrapping replicates. Finally, we rooted each ML tree at the midpoint using the ape and phangorn R packages [42, 43] and visualized these trees using iTOL (version 6.8.2) [44]. The homologous regions in other species and phylogenetic trees for non-redundant HGTs can be found in Table S13.

### Evaluation of the pipeline using simulated datasets

We constructed a simulated genome (called genome H) with 175 HGTs from a set of distantly related genomes (called Genome set D) to the human genome. Genome set D has 4 cruciferous plant genomes, including *Arabidopsis thaliana*, *Brassica napus*, *Brassica oleracea var. oleracea* and *Brassica rapa*), while Genome set C contains 4 primate genomes, *Pan paniscus*, *Pan troglodytes*, *Pongo abelii* and *Gorilla gorilla gorilla*. The 175 HGTs are sequences that have high similarity with genomes in Genome set D (> 90%) but have low similarity (< 50%) with genomes in Genome set C, the closely related group of genomes.

Firstly, the genome comparison between genomes in Genome set D was conducted using LASTZ [45] and Multiz [46] to obtain sequences whose identity in all genomes of Genome set D were > 90% and lengths > 200bps. These sequences were compared with the genomes in Genome set C and the sequences having low similarity (identity < 50%) were reserved. The obtained sequences were then clustered using the cd-hit-est program (version 4.6.6) [35] with minimum nucleotide identity set at 80%. The longest sequences from each cluster were selected as simulated HGTs, which were 175 in total. These 175 HGTs were then evenly divided into 10 groups according to their sequence lengths, and the copy numbers of which increased from 2^0^ to 2^9^ (Table S1). Eventually, 175 HGTs with different copy numbers were inserted into the human genome as genome H (data S2). Finally, we ran our pipeline with genome H as the target genome, genome set D as remote genome set, genome set C as closely related genome set and parameters M, N, L as 1, 1, 200 respectively. If the correct HGT region was covered more than 60% of its length by a predicted HGT region, the prediction was considered correct.

### Evaluation of the pipeline using reported whitefly HGTs

We acquired 131 HGTs using our pipeline with *Bemisia tabaci* as target organism and downloaded 170 HGTs previously reported in the whitefly [6]. HGTs identified by our pipeline were considered to be reported (BLASTN, matched length>135 bp and identity > 80%).

### Counting the copy numbers of HGTs

We run BLASTN [47] alignment for non-redundant HGT sequences against their host reference genomes, with the parameter “-e 1e-5”. For each HGT, we selected aligned regions that covered at least 80% of HGT regions with nucleotide identity > 80%. We then merged those aligned regions with overlapped coordinates. The copy number of each HGT was determined from the number of merged HGT copies.

### Comparison with reported HGTs in previous studies

We obtained reported HGTs for these model organisms from previous publications, including genomic coordinates and DNA sequences. HGTs in our study were considered novel if they did not match reported HGTs by genomic coordinates or sequence alignment (BLASTN, matched length > 135 bp and identity > 80%).

### Functional annotation of genes influenced by HGTs

Genome annotation files (GFF or GTF format) were obtained for model organisms from Ensembl(http://asia.ensembl.org) and Tair (https://www.arabidopsis.org), and they were used to identify protein-coding genes and non-coding genes likely to be affected by HGTs (overlapping with HGTs with at least 1 bp). Gene Ontology terms (GO terms) enrichment analysis of these genes were using the clusterProfiler R package [48] and agriGO [49] (FDR < 0.05).

### Validation of HGT regions in *Arabidopsis thaliana* with targeted PCR

Among the 23 HGTs related to membrane transport genes, 12 were considered as transferred from bacteria or fungi to plant. For these HGT regions, primer pairs are designed for the regions including the HGT regions and their 5,000 bp upstream and downstream regions (Fig. 5C) using Primer-BLAST [50]. Targeted PCR was executed to exclude the possibility of contamination. Agarose gel electrophoretic analysis was applied to judge whether PCR products were expected or not. If the PCR product matched our expected size, we then sequenced the PCR product using Sanger sequencing technology. Here, we considered that an HGT region was successfully validated if it was successfully amplified and its Sanger sequences were nearly identical to the corresponding regions of the genome sequence of *Arabidoposis thaliana*.

### Validation of HGTs with third-generation sequencing data

We downloaded third-generation sequencing data for the 10 representative species, except the lizard and frog from the SRA database and validated all the HGTs with third-generation sequencing data. We obtained 150 bp both upstream and downstream sequences for each HGT regions. Sequence alignment was done using minimap2 (version 2.14) [51] and the calculation of sequencing depth was done using samtools (version 1.16.1) [37] with default parameters. The sequencing depth of HGTs should not be significantly lower than that of 150 bp upstream and downstream sequences of HGTs (using student’s t test, *p* > 0.05). If an HGT region passed the above criteria in at least one sample, it was considered as validated successfully. The third-generation sequencing data information and validation results are shown in Table S18.

### 3D protein structure acquisition

We downloaded the structures of the HGT-related proteins, predicted by AlphaFold [52], from AlphaFold Protein Structure Database. Then we marked the HGT affected regions using PyMol (version 3.0.4) (http://www.pymol.org/pymol).

## Supplementary Information

Supplementary Material 1: Supplementary Figure 1. The HGT identification system for eukaryotes. Supplementary Figure 2. Evaluation of our HGT identification method. Supplementary Figure 3. The distribution of organisms in which HGT events occurred. Supplementary Figure 4. The length of overlapped regions between HGTs and genes. Supplementary Figure 5. Gene ontology (GO) functional enrichment for genes affected by HGTs. Supplementary Figure 6. The distribution of genetic elements affected by HGT regions with different copy numbers in 10 representative organisms. Supplementary Figure 7. Repeat characteristics of genomes of 10 representative organisms. Supplementary Figure 8. HGT examples overlapping with repeat sequences in 6 representative organisms. Supplementary Figure 9. Gene ontology (GO) functional enrichment for protein coding genes affected by HGTs not overlapping with repeat sequences. Supplementary Figure 10. HGT examples overlapping with CDS regions of protein coding genes in 7 representative organisms. Supplementary Figure 11. The phylogenetic tree of HGT region “NC_000009.12:131556125-131556269” involving human. Supplementary Figure 12. The phylogenetic tree of HGT region “NC_000075.7:103955726-103955938” involving mouse. Supplementary Figure 13. The phylogenetic tree of HGT region “NC_037329.1:133396645-133396784” involving cow. Supplementary Figure 14. The phylogenetic tree of HGT region “NC_048617.1:36292001-36292324” involving lizard. Supplementary Figure 15. The phylogenetic tree of HGT region “NC_030683.2:49997602-49997741” involving frog. Supplementary Figure 16. The phylogenetic tree of HGT region “NC_004354.4:14847937-14848199” involving fruit fly. Supplementary Figure 17. The phylogenetic tree of HGT region “NC_003070.9:18412013-18412370” involving Arabidopsis. Supplementary Figure 18. Full uncropped agarose gel electrophoresis image corresponding to Figure 5E. Supplementary Figure 19. The impact of parameter setting on the HGT recalling rate for the sequence composition-based screening step.

Supplementary Material 2: Supplementary Table 1. Grouping information of SG, CRG and DRG corresponding to different taxonomic levels of HGT in 10 representative species. Supplementary Table 2. Evaluation of the HGT identification pipeline on the simulated dataset. Supplementary Table 3. Evaluation of the HGT identification pipeline on HGTs reported previously. Supplementary Table 4. Detailed information of non-redundant HGTs, including the common ancestors of organisms within different self groups (SGs), the assembly ID and kingdom of organisms harboring these regions, their genomic copy numbers and the number of overlapping genes. Supplementary Table 5. The distribution of organisms in which HGT events occurred. Supplementary Table 6. HGT-appearance numbers between the 10 representative organisms and 1496 eukaryotes. Supplementary Table 7. Gene ontology (GO) functional annotation for genes affected by HGTs. Supplementary Table 8. Repetitive sequence composition of HGTs. Supplementary Table 9. Putative media of horizontal gene transfer of BovB retrotransposons and L1 retrotransposons. Supplementary Table 10. Examples of HGTs in cow, human, Arabidopsis, nematode, yeast and zebrafish genomes. Supplementary Table 11. The influence of HGTs not overlapping with repeat sequences on protein coding genes. Supplementary Table 12. Gene ontology (GO) functional annotation for genes affected by HGTs not overlapping with repeat sequences but overlapping with CDS regions of protein coding genes. Supplementary Table 13. Homologous regions in other species and phylogenetic trees for non-redundant HGTs. Supplementary Table 14. Gene ontology (GO) functional annotation for genes affected by HGTs using agriGO in Arabidopsis thaliana. Supplementary Table 15. A total of 23 genes (overlapping 21 HGT regions) associated with transmembrane transport in Arabidopsis thaliana. Supplementary Table 16. Comparison of secondary structure distributions between whole proteins and HGT regions. Supplementary Table 17. PCR validation of 12 genes affected by 11 HGT regions in Arabidopsis thaliana. Supplementary Table 18. Verification results of HGTs using third-generation sequencing data. Supplementary Table 19. Information of 10 representative organisms and other eukaryotes, bacteria, and viruses. Supplementary Table 20. Coverage matrices of WGS data for HGT homologous sequences in DRG.

Supplementary Material 3: Supplementary Data 1. Raw output of LASTZ alignment between 10 representative organisms with other eukaryotes (465GB). URL: 10.5281/zenodo.18005632. With a link on http://cgm.sjtu.edu.cn/hgt/data/Supplementary_Data_1.tar.gz due to large file size. Supplementary Data 2. The simulated genome (genome H) with 175 HGTs (2.9GB). URL: 10.5281/zenodo.18005632.

## Data Availability

All datasets generated and/or analyzed during the current study are available at [10.5281/zenodo.18005632], which can be accessed freely. All scripts, Supplementary Tables and an example of analysis pipeline application are also listed in the same webpage. All scripts used in this study are also available in GitHub at [https://github.com/SJTU-CGM/HGT.git].
