# Supplementary material for: Horizontal gene transfer is widespread in diverse eukaryotes
Source: BMC Genomics. 2026 Jun 8;27:661. doi: 10.1186/s12864-026-12958-7 (PMC13465657; doi:10.1186/s12864-026-12958-7)
Supplement: Supplementary file 1 [file 12864_2026_12958_MOESM1_ESM.zip › HGT_likun-Supplementaryfile.r4-submitted.docx]

Supplementary Materials for

**Horizontal gene transfer** **is widespread in diverse eukaryotes**

Kun Li^1$^, Fazhe Yan^1$^, Ziyao Feng^1^, Pingping Zhang^1^, Zhongqu Duan^1^, Qingqiu Gong^1^*, David L. Adelson^2^*, Chaochun Wei^1^*

*Corresponding author. Qingqiu Gong: [gongqingqiu@sjtu.edu.cn](mailto:gongqingqiu@sjtu.edu.cn), David Adelson: [david.adelson@adelaide.edu.au](mailto:david.adelson@adelaide.edu.au), Chaochun Wei: [ccwei@sjtu.edu.cn](mailto:ccwei@sjtu.edu.cn)

**This file includes:**

19 Figures.

**Other supporting materials for this manuscript include the following:**

19 Tables and 2 datasets.

Descriptions about the figures, tables and datasets are listed below. Supplementary tables are listed in a separate file, while supplementary tables and datasets are accessible from the given URL listed in the data availability.

**Supplementary Figure 1**

The HGT identification system for eukaryotes.

**Supplementary Figure 2**

Evaluation of our HGT identification method.

**Supplementary Figure 3**

The distribution of organisms in which HGT events occurred.

**Supplementary Figure 4**

The length of overlapped regions between HGTs and genes.

**Supplementary Figure 5**

Gene ontology (GO) functional enrichment for genes affected by HGTs.

**Supplementary Figure 6**

The distribution of genetic elements affected by HGT regions with different copy numbers in 10 representative organisms.

**Supplementary Figure 7**

Repeat characteristics of genomes of 10 representative organisms.

**Supplementary Figure 8**

HGT examples overlapping with repeat sequences in 6 representative organisms.

**Supplementary Figure 9**

Gene ontology (GO) functional enrichment for protein coding genes affected by HGTs not overlapping with repeat sequences.

**Supplementary Figure 10**

HGT examples overlapping with CDS regions of protein coding genes in 7 representative organisms.

**Supplementary Figure 11**

The phylogenetic tree of HGT region “NC_000009.12:131556125-131556269” involving human.

**Supplementary Figure 12**

The phylogenetic tree of HGT region “NC_000075.7:103955726-103955938” involving mouse.

**Supplementary Figure 13**

The phylogenetic tree of HGT region “NC_037329.1:133396645-133396784” involving cow.

**Supplementary Figure 14**

The phylogenetic tree of HGT region “NC_048617.1:36292001-36292324” involving lizard.

**Supplementary Figure 15**

The phylogenetic tree of HGT region “NC_030683.2:49997602-49997741” involving frog.

**Supplementary Figure 16**

The phylogenetic tree of HGT region “NC_004354.4:14847937-14848199” involving fruit fly.

**Supplementary Figure 17**

The phylogenetic tree of HGT region “NC_003070.9:18412013-18412370” involving *Arabidopsis*.

**Supplementary Figure 18**

Full uncropped agarose gel electrophoresis image corresponding to Figure 5E.

**Supplementary Figure 19**

The impact of parameter setting on the HGT recalling rate for the sequence composition-based screening step.

**Supplementary Table 1 (separate file).**

Grouping information of SG, CRG and DRG corresponding to different taxonomic levels of HGT in 10 representative species.

**Supplementary Table 2 (separate file).**

Evaluation of the HGT identification pipeline on the simulated dataset.

**Supplementary Table 3 (separate file).**

Evaluation of the HGT identification pipeline on HGTs reported previously.

**Supplementary Table 4 (separate file).**

Detailed information of non-redundant HGTs, including the common ancestors of organisms within different self groups (SGs), the assembly ID and kingdom of organisms harboring these regions, their genomic copy numbers and the number of overlapping genes.

**Supplementary Table 5 (separate file).**

The distribution of organisms in which HGT events occurred.

**Supplementary Table 6 (separate file).**

HGT-appearance numbers between the 10 representative organisms and 1496 eukaryotes.

**Supplementary Table 7 (separate file).**

Gene ontology (GO) functional annotation for genes affected by HGTs.

**Supplementary Table 8 (separate file).**

Repetitive sequence composition of HGTs.

**Supplementary Table 9 (separate file).**

Putative media of horizontal gene transfer of BovB retrotransposons and L1 retrotransposons.

**Supplementary Table 10 (separate file).**

Examples of HGTs in cow, human, *Arabidopsis*, nematode, yeast and zebrafish genomes.

**Supplementary Table 11 (separate file).**

The influence of HGTs not overlapping with repeat sequences on protein coding genes.

**Supplementary Table 12 (separate file).**

Gene ontology (GO) functional annotation for genes affected by HGTs not overlapping with repeat sequences but overlapping with CDS regions of protein coding genes.

**Supplementary Table 13 (separate file).**

Homologous regions in other species and phylogenetic trees for non-redundant HGTs.

**Supplementary Table 14 (separate file).**

Gene ontology (GO) functional annotation for genes affected by HGTs using agriGO in *Arabidopsis thaliana*.

**Supplementary Table 15 (separate file).**

A total of 23 genes (overlapping 21 HGT regions) associated with transmembrane transport in *Arabidopsis thaliana*.

**Supplementary Table 16 (separate file).**

Comparison of secondary structure distributions between whole proteins and HGT regions.

**Supplementary Table 17 (separate file).**

PCR validation of 12 genes affected by 11 HGT regions in *Arabidopsis thaliana*.

**Supplementary Table 18 (separate file).**

Verification results of HGTs using third-generation sequencing data.

**Supplementary Table 19 (separate file).**

Information of 10 representative organisms and other eukaryotes, bacteria, and viruses.

**Supplementary Table 20 (separate file).**

Coverage matrices of WGS data for HGT homologous sequences in DRG.

**Supplementary Data 1 (separate file).**

Raw output of LASTZ alignment between 10 representative organisms with other eukaryotes (465GB)

URL: <https://doi.org/10.5281/zenodo.18005632>

With a link on [http://cgm.sjtu.edu.cn/hgt/data/Supplementary_Data_1.tar.gz](http://cgm.sjtu.edu.cn/hgt/data/Supplementary_Data_1.tar.gztar) due to large file size.

**Supplementary Data 2 (separate file).**

The simulated genome (genome H) with 175 HGTs (3.1GB)

URL: <https://doi.org/10.5281/zenodo.18005632>

**Figure. S1.**

**The HGT identification system for eukaryotes.** Three main processes were involved: (A) Composition-based genomic fragments screening: split the chromosomes into fixed-size fragments and screen the fragments to get genome regions with the potential to harbor HGT regions according to k-mer frequencies; (B) Sequence alignment: sequence alignment between potential HGT containing fragments with the whole genomes of other organisms; (C) Validation using WGS datasets and phylogenetic tree: further screen the fragments using WGS datasets of selected organisms and re-screen the fragments based on the differences between the HGT region phylogenetic tree and the organism phylogenetic tree.

**Figure. S2.**

**Evaluation of our HGT identification method.** (A) Evaluation of our HGT identification pipeline on a simulated dataset. The X-axis represents the percentage of retained short sequences with top distances to the host genome in terms of sequence composition (k-mer), which were used in the consequent HGT event identification; and the Y-axis represents the HGT recall rate (the number of HGT events correctly predicted/the total number of HGT events in the simulated dataset), or the precision rate (the number of HGT correctly predicted/ the total number of HGT predicted). (B) Comparison of HGTs identified by our method and those reported in Li et al for whitefly [6]. A total of 131 HGTs identified by our method and 170 HGTs reported previously were compared. Of the 131 total HGTs identified by our method, approximately half (62 HGTs, ~47.3%) overlapped with those reported by Li et al. In the intersection, the numbers of overlapped HGT regions were reported (denoted as 62/55). The number 62 represents the count of HGTs predicted by our method, while 55 represents the corresponding count of HGTs reported by Li et al. For the 69 HGTs identified by our method only, more than half of them (36 HGTs) are not overlapping with protein coding regions and 24 of 33 HGTs overlapping with protein coding regions were overlapping less than 25% of their corresponding total protein. For the 115 HGTs reported but not predicted by our methods, 49 (~43%) were left in the first step of our method (choosing regions with top distances to the whole genome in terms of k-mer composition), 31 (~27%) had identity percentage lower than the threshold in our method for DRG, and 35 (~30%) had identity percentage higher than the threshold in our method for CRG.

**Figure. S3.**

**The distribution of organisms in which HGT events occurred.** The distribution of organisms that HGT events occurred in (A) 13 representative organisms, (B) Human, (C) Mouse, (D) Cow, (E) Lizard, (F) Frog, (G) Zebrafish, (H) Fruit fly, (I) Nematode, (J) Yeast and (K) *Arabidopsis*. The innermost ring is the kingdom to which organisms belong, the second outer ring is the phylum to which organisms belong, and the outermost ring is the class to which organisms belong. More information could be seen in Table S4.

**Figure. S4.**

**The length of overlapped regions between HGTs and genes.** The actual overlap lengths observed significantly exceed this 1bp threshold.

**Figure. S5.**

**Gene ontology (GO) functional enrichment for genes affected by HGTs.** Gene ontology (GO) functional enrichment for genes affected by HGT events in (A) Human, (B) Mouse, (C) Cow, (D) Fruit fly, (E) Yeast and (F) *Arabidopsis*.

**Figure. S6.**

**The distribution of genetic elements affected by HGT regions with different copy numbers in 10 representative organisms.** For each bar chart, the copy number of HGT regions is equal to 1, more than 1, more than 10, and more than 100 from left to right. (A) The distribution of genetic elements in all genes affected by HGT regions. Most of HGT regions overlapping with introns in vertebrates and fly had more than 100 copies. (B) The distribution of genetic elements in protein coding genes affected by HGT regions. (C) The distribution of genetic elements in noncoding genes affected by HGT regions.

**Figure. S7.**

**Repeat characteristics of genomes of 10 representative organisms.** LINE and SINE retrotransposons are two main transposable elements in vertebrates except frog and zebrafish, while DNA transposons are the most in frog, zebrafish and nematode**.** The proportion of LTR retrotransposons is highest in fruit fly, yeast and *Arabidopsis*.

**Figure. S8.**

**HGT examples overlapping with repeat sequences in 6 representative organisms.** The phylogenetic trees (left) represent the evolutionary relationship of all homologous sequences of this HGT region, and the plots (right) present sequence similarity between the homologous sequences from the model organism and the related species. The 6 examples were from six organisms: (A) Mouse, HGT region “NC_000076.7:36762503-36762679”, an HGT event between mammals and one protozoan *Babesia bigemina*; (B) Lizard, HGT region “NC_048613.1:69625593-69625731”, an HGT event between chordates and plants; (C) Frog, HGT region “NC_030684.2:134717620-134717765”, an HGT event between chordates and plants; (D) Zebrafish, HGT region “NC_007123.7:16653154-16653352”, an HGT event between chordates and one fungus *Puccinia striiformis f. sp. tritici*; (E) Fruit fly, HGT region “NT_033777.3:2464018-2464342”, an HGT event between insects and plants; and (F) Yeast, HGT region “NC_001141.2:209014-209163”, an HGT event between fungi and one plant *Juglans regia*. HGT examples overlapping with repeat sequences in the other 3 representative organisms can be found in main text. More information about these examples is provided in Table S13. **Figure. S9.**

**Gene ontology (GO) functional enrichment for protein coding genes affected by HGTs** **not overlapping with repeat sequences.** All HGT regions were not overlapping with repeat regions. Gene ontology (GO) functional enrichment for genes affected by HGT events in (A) Human, (B) Mouse, (C) Yeast and (D) *Arabidopsis*.
**Figure. S10.**

**HGT examples overlapping with CDS regions of protein coding genes in 7 representative organisms.** All HGT regions were not overlapping with repeat regions. The plots present sequence similarity between the homologous sequences from the model organism and the related species. The 7 examples were from seven organisms: (A) Human, HGT region “NC_000009.12:131556125-131556269”, an HGT event between chordates and bacteria, impacting gene *PRRT1B* which encoded proline rich transmembrane protein; (B) Mouse, HGT region “NC_000075.7:103955726-103955938”, an HGT event between chordates and bacteria, impacting gene *Acad11* associated with long-chain fatty acyl-CoA dehydrogenase activity; (C) Cow, HGT region “NC_037329.1:133396645-133396784”, an HGT event between chordates and bacteria, impacting gene *AKR7A2* which catalyzes the NADPH-dependent reduction of succinic semialdehyde to the endogenous neuromodulator, gamma-hydroxybutyrate; (D) Lizard, HGT region “NC_048617.1:36292001-36292324”, an HGT event between chordates and viruses, impacting gene *HSD3B7* encoding an enzyme which is involved in the initial stages of the synthesis of bile acids from cholesterol; (E) Frog, HGT region “NC_030683.2:49997602-49997741”, an HGT event between chordates and one protozoan *Plasmodium berghei ANKA*, impacting gene *bms1* which enabled GTP binding activity; (F) Fruit fly, HGT region “NC_004354.4:14847937-14848199”, an HGT event between insects and bacteria, impacting gene *Flo2* which enabled protease binding activity; and (G) *Arabidopsis*, HGT region “NC_003070.9:18412013-18412370”, an HGT event between plant and fungi, impacting gene *AT1G49750* which encoding Leucine-rich repeat (LRR) family protein. HGT examples overlapping with CDS regions of protein coding genes in other 3 representative organisms can be found in main text. More information about these examples is provided in Table S13.
**Figure. S11.**

**The phylogenetic tree of HGT region “NC_000009.12:131556125-131556269” involving human.** An HGT event between chordates and bacteria, impacting gene *PRRT1B* which encoded proline rich transmembrane protein.

**Figure. S12.**

**The phylogenetic tree of HGT region “NC_000075.7:103955726-103955938” involving mouse.** An HGT event between chordates and bacteria, impacting gene *Acad11* associated with long-chain fatty acyl-CoA dehydrogenase activity.
**Figure. S13.**

**The phylogenetic tree of HGT region “NC_037329.1:133396645-133396784” involving cow.** An HGT event between chordates and bacteria, impacting gene *AKR7A2* which catalyzes the NADPH-dependent reduction of succinic semialdehyde to the endogenous neuromodulator, gamma-hydroxybutyrate.
**Figure. S14.**

**The phylogenetic tree of HGT region “NC_048617.1:36292001-36292324” involving lizard.** An HGT event between chordates and viruses, impacting gene *HSD3B7* encoding an enzyme which is involved in the initial stages of the synthesis of bile acids from cholesterol.

**Figure. S15.**

**The phylogenetic tree of HGT region “NC_030683.2:49997602-49997741” involving frog.** An HGT event between chordates and one protozoan *Plasmodium berghei ANKA*, impacting gene *bms1* which enabled GTP binding activity.

**Figure. S16.**

**The phylogenetic tree of HGT region “NC_004354.4:14847937-14848199” involving fruit fly.** An HGT event between insects and bacteria, impacting gene *Flo2* which enabled protease binding activity.

**Figure. S17.**

**The phylogenetic tree of HGT region “NC_003070.9:18412013-18412370” involving *Arabidopsis*.** An HGT event between plant and fungi, impacting gene *AT1G49750* which encoding Leucine-rich repeat (LRR) family protein. **Figure. S18.**

**Full uncropped agarose gel electrophoresis image corresponding to Figure 5E.** The full uncropped agarose gel electrophoretic image of the PCR products of sequences including HGT regions transferred from bacteria or fungi to *Arabidopsis thaliana*. The cropped gel image shown in Figure 5E was derived from this full-length gel.

**Figure. S19.**

**The impact of parameter setting on the HGT recalling rate for the sequence composition-based screening step.** The parameters are k-mer size and the percentage of fragments with highest distances to that of the background genome. The X-axis represents the percentage of retained short sequences (0.5%, 1%, 2%, 5% ,10%,15% and 20% were tested), and the Y-axis represents the HGT recall rate (the number of HGT correctly predicted/ the total number of HGT reported in a previous study for the human genome). Here only 299 original reported human HGT regions remained after we had filtered out the candidate HGT regions containing more than 50% of simple repeats identified by TRF. When k=4, more than 80% of the 299 human HGT regions were identified by our pipeline if only 5% of the fragments with highest k-mer frequency distances to the human genome were input for the consequent analysis.
